# Analysis of the differences and influencing factors of rural population aging in Gansu Province—based on panel data from 2000 to 2022

**DOI:** 10.3389/fpubh.2025.1634712

**Published:** 2025-07-25

**Authors:** Yue Liu, Fengli Lv, Xiaochen Zhang, Jiancheng Wang

**Affiliations:** ^1^First Clinical Medical College, Gansu University of Chinese Medicine, Lanzhou, Gansu Province, China; ^2^Lanzhou University, Lanzhou, China; ^3^Gansu Provincial Hospital, Lanzhou, China

**Keywords:** population aging, panel data model, regional disparities, Gansu Province, influencing factors

## Abstract

**Objective:**

To analyze the spatial dimension differences and influencing factors of rural population aging in Gansu Province over the past 20 years.

**Methods:**

Data on population, economy, healthcare, and education from Gansu Province and its 14 cities (prefectures) from 2000 to 2022 were collected, with selected indicators quantified. A spatial dynamic panel data model was established to explore regional differences and the impact of influencing factors on population aging.

**Results:**

The aging of the rural population in Gansu Province continues to intensify, with regional disparities gradually widening. Among these, the central region exhibits the largest disparity and contribution rate, followed by the southeastern region, while the northwestern region shows the smallest. Intra-regional disparities, particularly in the central region, are the primary source of the overall disparities. Among the influencing factors, an increase in population density helps alleviate aging, whereas a rise in the illiteracy rate exacerbates population aging. Other factors, including the natural population growth rate, GDP *per capita*, and the number of hospital beds *per capita*, do not have a significant impact on population aging.

**Conclusion:**

The aging of the rural population in Gansu is intensifying, with regional disparities widening, influenced by multiple factors such as economic development, population growth, education levels, and medical resources.

## 1 Introduction

Population aging refers to the process in which the proportion of the older adults population aged 60 or 65 and above in the total population continues to increase. With declining fertility rates and increasing life expectancy, population aging has become a widespread social issue. According to data from the Seventh National Population Census, the aging rates in rural areas are 23.81 and 17.72%, which are 7.99 and 6.61% higher than those in urban areas, respectively, and have doubled compared to a decade ago. The aging of the rural population is occurring faster and more severely than in cities, drawing increasing attention from various sectors of society. For example, Lin et al. (1) analyzed the inter-provincial differences in rural population aging in China using the 31 provinces as the basic units; Zijuan et al. (2) examined the urban–rural disparities in population aging across the eastern, central, and western regions of China through provincial panel data; Xuefeng et al. (3) employed the grey relational analysis method to analyze the differences and influencing factors of rural population aging in the eastern, central, and western provinces of China. In existing research, studies on the differences in rural population aging across the country are relatively prominent, while research on underdeveloped regions, such as the northwest region, particularly Gansu Province, is scarce. This study aims to analyze the regional differences in rural population aging and their influencing factors in Gansu Province, providing a theoretical reference for addressing the issue of population aging in various regions of Gansu Province.

## 2 Materials and methods overview

### 2.1 Data sources

The data in this article are sourced from the “China Statistical Yearbook,” “Gansu Statistical Yearbook”, “Gansu Provincial Bureau of Statistics,” and the statistical bureaus of various cities (prefectures), selecting data from population censuses or sample surveys conducted in Gansu Province and its cities (prefectures) from 2000 to 2022.

### 2.2 Indicator selection

The older adults population aging coefficient (4) is the most commonly used indicator to measure the degree of population aging. It refers to the proportion of the population aged 65 and above in the total population of a certain region over a given period, reflecting the absolute degree of population aging. In this paper, the older adults population coefficient is selected as the explained variable. Given that there are many factors influencing population aging, they can be roughly categorized into four aspects: population development factors, educational development factors, economic development factors, and social factors, with secondary indicators established under each. Through literature review, the following indicators were ultimately selected to represent demographic indicators, educational indicators, economic indicators, health care, and social aspects respectively: Natural Population Growth Rate (NPGR, %), Illiteracy Rate (IR, %), Gross Domestic Product *per capita* (GDP, yuan), Number of Beds in Health Institutions per thousand people (NBHI), and Population Density (PD, people per square kilometer). The calculation of each indicator is performed after unifying the units of data for each year.

### 2.3 Research methodology

#### 2.3.1 Theil index

The Theil index (5) is a crucial metric for measuring income disparities between individuals or regions. This paper employs the Theil index to analyze the differences in the aging level of the rural population within and between regions in Gansu Province. Based on geographical location, Gansu Province is divided into three major regions: the northwest (Jiayuguan City, Jiuquan City, Zhangye City), the central region (Lanzhou City, Wuwei City, Dingxi City, Baiyin City, Jinchang City), and the southeast (Tianshui City, Qingyang City, Longnan City, Pingliang City, Gannan Prefecture, Linxia Prefecture). The disparities in rural population aging in Gansu Province are decomposed, and the overall differences, intra-regional differences, inter-regional differences, and related contribution rates among the 14 cities (prefectures) in Gansu Province are calculated. The calculation formula is as follows:


(1) $$T=\frac{1}{K}\sum_{q=1}^{\mathrm{Kq}}\{\frac{S_{q}}{\bar{S}}\times \ln\frac{S_{q}}{\bar{S}}\}$$

(2) $$T_{p}=\frac{1}{K_{p}}\sum_{q=1}^{K_{p}}\{\frac{S_{\mathrm{pq}}}{K}\times \ln\frac{S_{\mathrm{pq}}}{S_{q}}\}$$

(3) $$T=T_{w}+T_{b}=\sum_{p=1}^{3}\{\frac{K_{p}}{K}\times \frac{\bar{S_{p}}}{\bar{S}}\times T_{p}\}+\sum_{p=1}^{3}\{\frac{K_{p}}{K}\times \frac{\bar{S_{p}}}{\bar{S}}\times \ln\frac{\bar{S_{p}}}{\bar{S}}\}$$

In Equation 1, T represents the overall Theil index of the rural population aging level in Gansu Province. The smaller the Theil index, the smaller the overall disparity in the rural population aging level, and vice versa. q denotes the city (prefecture), k represents the number of cities (prefectures), $S_{q}$ represents the rural population aging level of the city (prefecture), and $\bar{S\;}$ represents the average rural population aging level in Gansu Province. In Equation 2, $T_{p}$ represents the rural population aging level of region p, $K_{p}$ represents the rural population aging level of region p, $S_{\mathrm{pq}}$ represents the rural population aging level of region p, represents the number of cities (prefectures) in region p, $\bar{S_{p}}$ and represents the average rural population aging level in Gansu Province. In Equation 3, the rural population aging level is further decomposed into the intra-regional Theil index $T_{w}$ and the inter-regional Theil index $T_{b}$ . Additionally, define $T_{w}∕T$ and $T_{b}∕T$ as the contribution rates of intra-regional differences and inter-regional differences to the overall differences, respectively, $\left(S_{p}∕S)\times (T_{p}∕T\right)$ as the contribution rate of each region to the overall intra-regional differences, $S_{p}$ represents the sum of the rural population aging levels in each city (prefecture) within region p, and S represents the sum of the rural population aging levels in Gansu Province.

#### 2.3.2 Panel data

Panel data (6) is a type of two-dimensional data, which involves taking multiple cross-sections over a time series and selecting sample observations on these cross-sections. Ultimately, panel data consists of observations from multiple individuals and multiple time periods. The general form of a panel data model is as follows:


$$y_{\mathrm{it}}=a_{\mathrm{it}}+\sum_{k=1}^{k}\beta_{\mathrm{kit}}X_{\mathrm{kit}}+\mu_{\mathrm{it}}\;i=1,2,\cdots ,n\;t=1,2,\cdots ,T$$

$a_{\mathrm{it}}$ is the intercept term, $y_{\mathrm{it}}$ representing the observed value of the explanatory variable for an individual i at time t . This study is on the value of the older adults population coefficient for region i in year t . $X_{\mathrm{kit}}$ refers to the observed value of the k -th explanatory variable for individual i at time $t.\beta_{\mathrm{kit}}$ represents the parameter to be estimated for the k -th explanatory variable, and $\mu_{\mathrm{it}}$ is the random error term. Based on the different estimations of the parameters to be evaluated and the random error terms, panel data models are divided into fixed effects models, mixed effects models, and random effects models. Typically, the Hausman test is used to determine whether a fixed effects model or a random effects model is appropriate, while the F test is used to decide between a fixed effects model and a mixed effects model. Theil index calculations and panel data regression analysis are performed using Stata15.0. Due to missing data in some areas, multiple imputation is employed to handle the gaps and minimize the impact on the analysis results.

## 3 Results

### 3.1 Measurement and decomposition of regional disparities in rural population aging in Gansu Province

#### 3.1.1 Overall discrepancy measurement

The Theil Index was used to measure the aging index of the rural population in 14 cities (prefectures) of Gansu Province from 2000 to 2022. As shown in Table 1, the results indicate that the Theil Index of the aging level of the rural population in Gansu Province generally exhibited a fluctuating upward trend from 2000 to 2022, increasing from 0.5162 in 2000 to 1.7668 in 2022, suggesting that regional disparities in the aging degree of rural areas in Gansu Province have gradually widened. During the observation period, the years 2005 (2.0535), 2016 (1.5989), and 2022 (1.7668) were notably higher, serving as critical junctures for the expansion of disparities, which may be closely related to population mobility, uneven economic development, or policy adjustments. From the perspective of the contribution rate of inter-regional differences, the rate was relatively low in the early period (2000–2003), with the primary differences stemming from intra-regional disparities. However, after 2004, the contribution of inter-regional differences increased, exceeding 40% in multiple years (such as 2002, 2005, 2007, and 2022), indicating that the uneven distribution of rural aging levels among the cities and prefectures within Gansu Province gradually became apparent.

**Table 1:** Theil index and contribution rate of rural aging level in Gansu Province and 14 cities (prefectures) from 2000 to 2022—three major regions.

Year	Overall difference	Inter-regional differences and contribution rates	Intra-regional disparities and their contribution rates			
			Overall	Northwest	Central	Southeast
2000	0.5162	0.0527 (10.21)	0.4635 (89.79)	0.3783 (2.53)	0.6159 (32.86)	0.3923 (52.44)
2001	1.0030	0.2989 (29.80)	0.7041 (70.20)	0.5844 (3.21)	0.7875 (57.33)	0.4512 (9.66)
2002	1.1528	0.6553 (56.85)	0.6553 (56.85)	0.2187 (1.08)	0.7176 (52.33)	0.3868 (3.44)
2003	0.5670	0.0815 (14.38)	0.4855 (85.62)	0.3189 (5.83)	0.5265 (35.33)	0.4823 (43.89)
2004	0.8093	0.1604 (19.82)	0.6489 (80.18)	0.3761 (2.46)	0.8434 (62.00)	0.3612 (15.72)
2005	2.0535	0.8196 (39.91)	0.1234 (60.09)	0.0046 (0.00)	1.2769 (59.33)	0.4021 (0.76)
2006	1.1512	0.2920 (25.37)	0.8592 (74.63)	0.0000 (0.00)	0.9942 (70.03)	0.6636 (5.25)
2007	1.7275	0.7566 (43.80)	0.9709 (56.20)	0.0252 (0.01)	1.0199 (55.21)	0.3073 (0.98)
2008	1.5721	0.5229 (33.26)	1.0492 (66.74)	0.0354 (0.10)	1.2289 (66.57)	0.0112 (0.07)
2009	0.0945	0.0225 (23.78)	0.0720 (76.22)	0.0637 (11.72)	0.0569 (17.60)	0.0829 (46.90)
2010	0.5641	0.1269 (22.49)	0.4372 (77.51)	0.0005 (0.01)	0.0026 (0.10)	0.6435 (77.40)
2011	0.0054	0.0013 (23.99)	0.0041 (76.01)	0.0001 (0.46)	0.0072 (48.89)	0.0033 (26.57)
2012	0.5179	0.1376 (26.57)	0.3803 (73.43)	0.0022 (0.05)	0.6144 (73.03)	0.0068 (0.34)
2013	0.0107	0.0027 (25.49)	0.0080 (74.51)	0.0041 (7.48)	0.0124 (45.68)	0.0055 (21.29)
2014	0.0051	0.0016 (30.47)	0.0036 (69.53)	0.0001 (0.41)	0.0064 (46.30)	0.0027 (22.29)
2015	0.0089	0.0021 (23.82)	0.0068 (76.18)	0.0054 (11.57)	0.0096 (41.29)	0.0049 (23.39)
2016	1.5989	0.5397 (33.75)	1.0592 (66.25)	0.0099 (0.03)	1.2325 (66.20)	0.0033 (0.02)
2017	1.3018	0.4175 (32.07)	0.8844 (67.93)	0.0010 (0.00)	1.1010 (67.90)	0.0029 (0.03)
2018	0.0087	0.0023 (26.13)	0.0064 (73.99)	0.0014 (3.06)	0.0121 (54.33)	0.0034 (16.58)
2019	0.0023	0.0006 (23.93)	0.0018 (76.07)	0.0012 (10.70)	0.0011 (17.13)	0.0027 (48.33)
2020	0.0056	0.0002 (3.74)	0.0054 (96.26)	0.0032 (12.21)	0.0018 (11.90)	0.0018 (13.64)
2021	0.0072	0.0003 (4.29)	0.0069 (95.71)	0.0042 (12.28)	0.0028 (14.14)	0.0120 (69.29)
2022	1.7668	0.8229 (46.58)	0.9439 (53.42)	0.9982 (45.50)	0.0015 (0.00)	0.8948 (7.92)

#### 3.1.2 Regional disparity decomposition

The Theil indices of rural population aging and the contribution rates of various Theil indices within and between the three major regions divided by geographical location in the northwest, central, and southeast of Gansu Province were calculated. From the further decomposition results of intra-regional differences, the average Theil indices and contribution rates of rural population aging levels in the northwest, central, and southeast of Gansu Province from 2000 to 2022 were 0.1320, 0.4814, and 0.2230, and the average contribution rates were 5.68, 43.28, and 22.03%, respectively. The internal disparities within the northwestern region are generally small, with their contribution rate mostly below 10% in most years, indicating that the rural aging levels in this area are relatively close and exhibit a certain consistency. In contrast, the internal disparities in the central region are more pronounced and constitute the primary source of regional differences across the province. In some years, such as 2006, 2016, and 2022, the contribution rate of internal disparities exceeded 65%, highlighting significant urban–rural development gaps within the central region and notable variations in the degree of rural aging across different cities and prefectures. The internal disparities in the southeastern region were relatively large in the early years, with a contribution rate reaching 52.44% in 2000, but have shown an overall declining trend in recent years, dropping to 0.89482 in 2022, which suggests a convergence in aging levels within the southeastern region.

### 3.2 Analysis of influencing factors on regional disparities in rural population aging in Gansu Province

#### 3.2.1 Results of variable correlation analysis

The analysis results are shown in Table 2. According to the correlation analysis results presented in the table below, the explanatory variables selected in this study—natural population growth rate (NPGR), number of beds in health institutions (NBHI), and population density (PD)—exhibit significant correlations with population aging (PC) at the 10% significance level. The explanatory variables all show negative correlations with population aging (PC). GDP *per capita* (GDP) and population density (PD) demonstrate a significant positive correlation at the 5% level, indicating a mutually positive relationship between GDP *per capita* and population density (PD).

**Table 2:** Correlation analysis between population aging and various factors.

Indicator	PC	NPGR	GDP	NBHI	PD
PC	1.0000				
NPGR	−0.0378**	1.0000			
GDP	−0.1038	−0.3965	1.0000		
NBHI	−0.0257**	−0.4655	0.6034	1.0000	
PD	−0.0289**	0.1570	0.0967*	0.0136**	1.0000

** and * indicate significance at the 5 and 10% levels, respectively (due to missing illiteracy rates in various cities and prefectures, a separate analysis will be conducted subsequently).

#### 3.2.2 Stationarity test

To avoid generating spurious regression results, it is necessary to conduct a stationarity test on the data, specifically a unit root test. In this study, the LLC (Levin-Lin-Chu) method and the ADF (Augmented Dickey-Fuller) method were selected for the unit root test. As shown in Table 3, the unit root test results for population aging (PC), number of beds in health institutions (NBHI), population density (PD), and *per capita* GDP (GDP) are all stationary, whereas the test result for natural population growth rate (NPGR) is non-stationary. After performing first-order differencing on NPGR and conducting the stationarity test again, the result becomes stationary.

**Table 3:** Unit root test.

Indicator	LLC test			ADF-Fisher test			Stationarity test
	t-value	Adjusted t-value	*p*-value	t-value	Adjusted t-value	*p*-value	
PC	−82.6870	−22.5186	0.0000	99.9394	9.0291	0.0000	Stationary
NPGR	−3.9371	4.5168	1.0000	3.0287	−3.4820	1.0000	Non-stationary
ΔNPGR	−10.8881	−1.4539	0.0730	166.1067	17.5713	0.0000	Stationary
GDP	−7.5835	0.1928	0.5764	67.4115	4.8298	0.0000	Stationary
NBHI	−9.2269	−5.3646	0.0000	70.2792	5.2000	0.0000	Stationary
PD	−4.5231	−7.9328	0.0000	128.4556	12.7106	0.0000	Stationary

*p* < 0.05 is significant.

#### 3.2.3 Panel model regression

Based on the different estimations of the parameters to be estimated and the random error terms, panel data models are divided into fixed effects models and random effects models. Typically, the Hausman test is used to determine whether a fixed effects model or a random effects model is more appropriate. As shown in Table 4, the *p*-value is 0.0549. Using a 5% significance level, since the *p*-value is greater than 0.05, the null hypothesis H0 is accepted, which states that all μit are uncorrelated with the explanatory variables. Therefore, in conclusion, the random effects model should be used (see Table 5).

**Table 4:** Hausman test results.

Indicator	Random effects model	Fixed effects model	Coefficient difference	Standard error of coefficient difference
ΔNPGR	0.0414	0.0409	0.0005	-
NBHI	0.0139	0.0697	−0.0558	0.0189
PD	−0.0306	−0.0157	−0.0149	0.0054
GDP	2.13 × 10^−6^	−8.07 × 10^−6^	0.0000	3.87 × 10^−6^

**Table 5:** Random effects panel data regression results.

Indicator	Regression coefficient	Standard error	Z-value	*P*-value	Statistical significance
ΔNPGR	0.0409	0.0736	0.56	0.578	Not significant
NBHI	0.0697	0.0673	1.04	0.300	Not significant
PD	−0.0157	0.0056	−2.81	0.005	5% significant
GDP	−8.07 × 10^−6^	8.49 × 10^−6^	−0.95	0.342	Not significant
Constant term	2.8322	0.9742	2.91	0.004	1% significant

Based on the random effects regression results, the following regression model can be obtained:


$$\begin{array}{l} PC_{i,t}=0.0409\text{ΔNPG}R_{i,t}-8.07\times 10^{-6}\mathrm{GD}P_{i,t}\\ +0.0697\mathrm{NBH}I_{i,t}-0.0157PD_{i,t}+2.8322 \end{array}$$

As can be seen from the results, population density (dp) has a significant negative impact on population aging, with a regression coefficient of −0.0157, which is significant at the 5% level. This indicates that regions with higher population density have lower levels of aging, possibly related to population mobility and the concentration of younger populations in cities. Other variables such as the natural population growth rate, the number of beds in health institutions, and *per capita* GDP did not pass the significance test, indicating that under the current model settings, these factors do not have a significant impact on population aging. The overall goodness-of-fit R^2^ of the model is 0.0021. Although the value is relatively low, it is quite common in panel data analysis in the field of population sociology. The proportion of individual effects to total error is 0.6874, indicating significant regional differences, and thus the adoption of a random effects model is justified.

#### 3.2.4 Supplementary analysis: OLS regression analysis of illiteracy rate on population aging

This study selects the illiteracy rate (%) from the census statistics for analysis. Currently, only the data from the 5th (2000), 6th (2010), and 7th (2020) national population censuses for 14 prefectures and cities are available, with illiteracy rate data missing for other years. Due to the characteristics of the data, OLS regression (Ordinary Least Squares) is employed for analysis. Given that the data sources for the illiteracy rate and the natural population growth rate are from the national population censuses and the time points are limited (only 2000, 2010, and 2020), to preserve the data structure and explanatory power, this study uses the original variables for modeling without first-order differencing. The results are shown in Table 6.

**Table 6:** OLS regression analysis results.

Indicator	Regression coefficient	Standard error	t-value	*P*-value	Statistical significance
IR	0.2648	0.1213	2.18	0.036	Significance
NPGR	−0.1967	0.2164	−0.91	0.370	Not significant
Indicator	Regression coefficient	Standard Error	t-value	*P*-value	Statistical significance
NBHI	−0.6217	0.0004	−2.28	0.029	Significance
PD	−0.0032	0.0049	−0.66	0.513	Not significant
GDP	−2.47 × 10^−6^	0.0000	−0.01	0.991	Not significant
Constant term	4.0404	2.5208	1.60	0.118	Not significant

*p* < 0.05 is significant.

The coefficient of determination R^2^ for this regression model is 0.3811, indicating that the model can explain approximately 38.11% of the variation in population aging (pc). This means that the five variables—illiteracy rate (IR), natural population growth rate (NPGR), number of beds in health institutions (NBHI), population density (PD), and GDP *per capita*—can collectively account for the differences in population aging to some extent. However, approximately 62% of the variation is influenced by other variables not included in the model, suggesting that the model’s explanatory power is moderately low. The F-statistic of the model is 3.26, with a corresponding significance level (Prob > F) of 0.016, which is less than 0.05, indicating that the overall regression model is statistically significant. This means that at least one explanatory variable in the model has a significant impact on the dependent variable, population aging. The regression coefficient for the illiteracy rate (ir) is 0.2648, with a *p*-value of 0.036, which is significant at the 5% level. This suggests that, holding other variables constant, an increase of one unit in the illiteracy rate will lead to an approximate rise of 0.26 units in the level of population aging, indicating a significant positive correlation between the illiteracy rate and the degree of aging. The regression coefficient for the number of beds in health institutions (NBHI) is −0.6217, with a *p*-value of 0.029, which is significant at the 5% level. This indicates that, holding other variables constant, for every one-unit increase in the number of beds in health institutions, the level of population aging decreases by approximately 0.62 units.

## 4 Discussion

### 4.1 Regional decomposition and expansion trends of rural population aging in Gansu Province

This study measures and decomposes the regional disparities in rural population aging in Gansu Province based on the Theil index. The results show that from 2000 to 2022, the overall disparity in rural population aging among the 14 cities (prefectures) in Gansu Province has shown a continuous expansion trend, with the Theil index rising from 0.5162 to 1.7668, reaching its peak in 2022, indicating significant differences in rural aging levels among regions. Further decomposition of regional disparities reveals that the rural population aging disparity in the central region of Gansu Province is the most prominent, with both the Theil index and its contribution rate ranking first among the three major regions, echoing the phenomenon of aging disparities on both sides of the “Hu Huanyong Line” found in the study by Chen et al. (7), indicating that population density boundaries have a significant impact on the distribution of aging. The average contribution rate in the central region is 43.28%, in the southeast region is 22.03%, and in the northwest region is only 5.68%. This aligns with the spatial characteristics of “higher in the east and lower in the west” in rural aging in China as shown in existing research (8). This reflects the uneven development within the central region of Gansu Province, where the urban–rural gap is significant, making it the primary area driving the expansion of rural aging disparities across the province. The reasons for these regional differences may include: significant disparities in rural economic development levels among counties in the central region, with notable impacts of population migration on the aging structure; while the southeast region is influenced by the factors of ethnic minority settlements, where the aging process is intertwined with cultural and reproductive traditions; the northwest region, due to its smaller population base, experiences a relatively slower pace of aging development. In line with the recommendations for research on the Shaanxi-Gansu region (9), which suggest “the need to formulate differentiated aging policies for different regions (such as sparsely populated areas).” To address the increasing rural aging in central Gansu due to urban–rural disparities, a targeted, regionally specific, coordinated development strategy is crucial. This requires equalizing public services, reallocating healthcare and eldercare resources, and enhancing primary-level service delivery. First, advancing the parity of public services across urban and rural settings is essential, with emphasis on reallocating healthcare and eldercare infrastructure and improving the service delivery capacity at the primary level. Second, policies should incentivize youth to return and engage in local entrepreneurship, thereby reducing demographic outflow via industrial policy support and reinforcing community-based older adults care systems. Third, county-level aging profiles should inform the design of targeted interventions, with preferential fiscal allocations directed to regions facing accelerated aging and constrained resource availability. To improve well-being and engagement for rural older adults, we should expand culturally appropriate eldercare and mutual aid systems like time banking. Additionally, an aging surveillance database and digital eldercare platform will enable targeted service delivery and data-driven policy. Furthermore, establishing an aging surveillance database alongside a digital eldercare platform is necessary to enable precision service delivery and data-informed policy governance.

### 4.2 Population density has a significant negative impact on the aging of the rural population in Gansu Province

This study is based on panel data from 14 prefectures and cities in Gansu Province from 2000 to 2022, employing a random effects model to analyze the impact of population density on the level of population aging. The results show that the regression coefficient of population density is −0.0157, which is significant at the 5% level, indicating that for every unit increase in population density, the level of population aging will correspondingly decrease. This result is similar to the study on the influencing factors of rural population aging differences in Anshun City, Guizhou Province (10), suggesting that there may be a non-linear relationship between population density and the distribution of aging. It corroborates the findings of Yuan et al. (11), whose research revealed non-linear differences in the effects of aging between urban and rural areas, specifically that the marginal effect increases after urban areas cross the aging threshold, whereas the opposite is true for rural areas. Cities generally possess greater population attractiveness and employment opportunities (12). To address the impact of population density on aging, it is essential to promote the construction of urban clusters to facilitate population agglomeration, optimize rural industrial structures to attract the return of young people, implement differentiated regional policies, strengthen the allocation of older adults service resources in high-density areas, improve the urban–rural employment linkage mechanism and fertility support system, and establish a gradient older adults care network that integrates medical services and older adults care.

### 4.3 *Per capita* GDP, number of beds in health institutions, and natural population growth rate have no significant impact on the aging of rural population in Gansu Province

The regression results of this study indicate that the impact of *per capita* GDP and the number of beds in health institutions on population aging is not significant. Although the regression coefficient of the natural population growth rate is negative, it also did not pass the statistical significance test. This suggests that, under the current real-world context of rural areas in Gansu Province, the direct correlation between the level of economic development and the aging process remains unclear. Firstly, while *per capita* GDP can reflect the regional economic development level to a certain extent, its mechanism of influence on rural population aging is relatively complex. Economic advancement has, on one side, contributed to higher average educational attainment, greater female labor force participation, and increased opportunity costs associated with childbearing and parenting. These shifts have collectively driven down fertility rates and stimulated population out-migration, thereby accelerating demographic aging (13, 14). On the other side, economic growth has also enabled expanded investment in public services—particularly in healthcare and eldercare—which in turn improves infrastructure and facilitates more equitable distribution of health resources, enhancing rural aging support systems and potentially mitigating some aging-related challenges (15). These opposing forces may neutralize each other in statistical models, rendering the net regression effect insignificant. Notably, the co-occurrence of these “accelerating” and “buffering” mechanisms presents marked spatial disparities. In the central region, resource allocation imbalances and uneven development amplify the fertility-suppressing effects more than improvements in old-age care, deepening the aging trajectory. In contrast, southeastern areas—shaped by ethnic cultural dynamics and policy support—exhibit more intricate pathways in the aging-economy nexus. Meanwhile, northwestern regions, constrained by underdeveloped economies, display muted responses from both mechanisms. Secondly, the number of beds in health institutions, as an important indicator for measuring the supply of medical resources, should theoretically provide certain support to population aging. However, in rural areas, the accessibility and utilization efficiency of medical resources are often lower than in cities (16). Coupled with the fact that the majority of the older adults population tends to seek medical care at the grassroots level (17), changes in the number of institutional beds may not directly translate into improved services for the older adults, thus potentially having a weaker impact on aging. Although *per capita* GDP and the number of hospital beds represent macro-level indicators of economic development and healthcare capacity, respectively, they do not show statistically significant associations with population aging in this study. This may be attributed to limitations in sample selection or the operational definitions of the variables. While *per capita* GDP indicates general regional prosperity, it fails to capture the specific economic security experienced by the rural older adults. Similarly, the total number of hospital beds tends to reflect aggregate healthcare infrastructure rather than the accessibility, quality, or appropriateness of eldercare services. Consequently, such macro indicators may inadequately represent the true determinants influencing aging trends within regression frameworks. To better understand socioeconomic factors in rural aging, future studies should examine the dependency ratio, pension coverage, and access to home-based care. Additionally, although the natural population growth rate (NGR) is theoretically negatively correlated with aging—meaning that a lower growth rate corresponds to a higher degree of aging—its impact may not have reached a significant level due to the overall low NGR within the province during the sample period, limited variations among cities and prefectures, and the temporal discontinuities and precision limitations of the statistical data. This situation may also be related to China’s entry into a deeply aging society (18), which has led to more severe infertility issues, confirming the correlation between the decline in natural population growth and the intensification of aging. To prevent aging from becoming the dominant criterion in healthcare resource allocation, it is imperative to transition from an “age-based” to a “health needs-based” distribution framework, thereby promoting a more equitable and evidence-informed healthcare delivery system. On one hand, a tiered protection mechanism should be established, guided by disease burden and functional capacity, to facilitate the rational distribution of medical resources across diverse populations, including older adults, children, women, and younger individuals with chronic illnesses. Concurrently, attention must be paid to the multifaceted influences of economic conditions, healthcare infrastructure, and natural demographic trends on population aging. Efforts should focus on upgrading rural specialty industries, broadening local employment opportunities for young people, and curbing population outmigration. Furthermore, regional medical alliances must be reinforced to enhance the diagnostic and treatment capabilities of primary care institutions and to build an integrated older adults care system that encompasses home-based, community-based, and institutional services, thereby advancing the integration of medical care and health maintenance. On the other hand, accelerating urban–rural infrastructure integration and establishing talent mobility mechanisms are essential to achieving balanced regional development in both economic and health sectors, ultimately addressing the structural drivers of aging. In parallel, reforms in healthcare payment models should be pursued, including the implementation of risk-adjusted reimbursement mechanisms to enable dynamic and needs-based resource distribution. Additionally, the development of smart healthcare systems and big data platforms should be promoted to enhance service delivery efficiency and the precision of resource allocation. Through the synergistic advancement of policy, service innovation, and digital technologies, the potential “crowding-out effect” of aging on health resource allocation can be mitigated, thereby ensuring the fair, efficient, and sustainable use of healthcare resources.

### 4.4 The illiteracy rate has a significant positive impact on the aging of the rural population in Gansu Province

In the OLS regression analysis of data from the fifth (2000), sixth (2010), and seventh (2020) national population censuses, the illiteracy rate showed a significant positive impact on population aging. The regression results indicated that the regression coefficient for the illiteracy rate was 0.2648, with a *p*-value of 0.036, suggesting that for every one percentage point increase in the illiteracy rate, the level of population aging rises by approximately 0.265 percentage points. The findings imply that education level plays a crucial moderating role in population aging. The illiteracy rate, representing a key barrier in both cultural and educational dimensions, has been recognized as a contributing factor to frailty persistence in older adults (19). It restricts seniors’ ability to acquire and interpret health-related information, which in turn compromises disease self-management and adherence to health-promoting behaviors (20). Low health literacy, often associated with illiteracy, may diminish the effectiveness of informal caregiving and caregivers’ ability to provide adequate support (21). In the sociocultural context of China, intergenerational support plays a vital role in addressing population aging, with financial assistance, caregiving, and emotional communication significantly enhancing both mental and physical health among older adults (22). However, the strength of this support is shaped more by filial piety norms, the quality of interpersonal communication within families, and the level of involvement by children, rather than being directly determined by literacy levels. Research in China has found (19) that education level significantly positively affects the health status of the older adults, and low education levels are more prone to insufficient health literacy (23). A study in China on the accessibility disparities in the spatial distribution of resources in Xiamen (24) revealed that areas with high illiteracy rates often face imbalanced distribution of older adults care resources, while the improvement of education levels contributes to enhancing the efficiency of resource utilization (25). Regions with higher illiteracy rates may be accompanied by lower fertility rates, poorer health conditions, and insufficient older adults care resources (24, 26), thereby exacerbating the issue of population aging. When formulating policies to address population aging, educational factors should be prioritized in regional resource allocation. To promote the optimization of population structure, it is advisable to strengthen investments in educational infrastructure, particularly in regions where illiteracy rates remain high. Priority should be given to expanding access to foundational education and lifelong learning facilities, with a focus on implementing tailored programs for older adults, including literacy improvement, health education, and digital competency training. These efforts aim to enhance health self-management and increase social engagement among the older adults. Concurrently, educational inequalities must be factored into the planning of care services, ensuring that resource distribution aligns with the actual needs of older individuals with limited formal education. Such inclusive eldercare strategies promote diversity and equity, improving well-being in underserved areas and reducing the societal burden of aging. Therefore, eliminating economic barriers is a crucial foundation for reducing illiteracy rates, but it requires comprehensive strategies to achieve sustainable results. The primary measure is to implement universal free education—waiving tuition fees and textbook costs—complemented by targeted economic support such as school meal programs, transportation subsidies, and direct cash transfers to families in need. These measures help alleviate the financial burden of schooling. Secondly, it is necessary to enhance teacher training and reform practical curricula. Finally, at the systemic level, community mobilization and data monitoring must be strengthened. It is also essential to integrate economic, cultural, and quality dimensions by eliminating cultural barriers and mitigating the impact of economic disadvantages on literacy outcomes. Beyond formal education, health education is another crucial domain closely linked to healthy aging. Health education should be systematically integrated into community health systems, focusing on improving health literacy and self-care skills across the life course. Regular training, local health education centers, and age-friendly communication strategies should be established. Moreover, health policies should embed educational components by incorporating preventive and health management services into basic insurance schemes. This will support a full-chain model of education, prevention, and care. Through coordinated educational and health interventions, these strategies aim to reduce health disparities, enhance functional ability, and promote healthy aging across diverse population groups.

## 5 Limitations and prospects of the research

The model in this study exhibits a low level of fit, indicating that the explanatory variables have limited explanatory power over aging. Future research could further introduce variables such as social security, education expenditure, and the proportion of the floating population. Moreover, the data consists of provincial and municipal panels and cannot be refined to the micro-family level. Subsequent research could combine micro-survey data for a more in-depth mechanism analysis.

## Data Availability

The original contributions presented in the study are included in the article/supplementary material, further inquiries can be directed to the corresponding author.
